# Medical and Philosophical News

**Published:** 1781-06

**Authors:** 


### SECTION III.

### Medical and Philosophical News.

THE Society of Sciences at Fluſhing have propoſed the following prize queſtions, viz. "What are the real cauſes and ſymptoms "of the fevers that prevail during the autum- "nal months in the garriſoned towns of Dutch "Flanders? and what are the beſt means to "be employed for preventing and curing them, "eſpecially among the troops?" The prize is to to be a gold medal, and the diſſertations are to be written in Latin, French, or Flemiſh, and ſent to Mr. Juſte Tjeenk, ſecretary to the ſociety, on or before Jan. 1, 1782.

---

Hitherto but little ſatisfactory information has been derived from meteorological diaries. It is probable, however, that by comparing obſervations of this kind made in a great number of different places at the ſame time, ſome uſeful facts might be eſtabliſhed. It is with pleaſure, therefore, we inform our readers, that ſeveral ſocieties have lately been inſtituted for this purpoſe. One of theſe is at Carlſruhe, under the patronage of the Margrave of Baden-Dourlach. Profeſſor Brökman is appointed director of this new eſtabliſhment, and the neceſſary obſervations are to be made at ſixteen different places in the territories of Baden.—The Elector Palatine, it ſeems, has ſince adopted a ſimilar plan in his dominions; and at the Hague, a medico-meteorological ſociety has lately been eſtabliſhed with the ſame views.

---

Mr. James Kerr, ſurgeon in Bengal, to whom the public are already indebted for a deſcription of of the tree producing the *terra japonica*, inserted in the Med. Obs. & Inq. Vol. V. has lately transmitted to the Royal Society a curious account of the insect that produces gum lacca.

---

We are informed that M. Andry, an ingenious physician at Paris, has lately given the inspissated juice of the *saponaria officinalis*, or *sopewort*, to a great number of patients labouring under gonorrhœa. The patients took about half an ounce of this medicine daily, and in general, a cure was effected in about a fortnight without the assistance of any other remedy.

---

In the winter of 1779-80, Mr. Patrick Wilson of Glasgow made some experiments, by which it appeared that the surface of snow was in general colder, and often by many degrees, than either the air above it, or the body of the snow underneath. Various hypotheses were formed by the ingenious experimenter and his friends, to account for this difference. Evaporation, as it seemed the most natural, first occurred. But other circumstances led Mr. Wilſon to ſuſpect that it was occaſioned by the air giving out and depoſiting its hoar-froſt. This extraordinary fact was communicated to the Royal Society, and has ſince appeared in the Philoſophical Tranſactions. In another paper lately preſented to the Society, Mr. Wilſon relates ſome farther experiments which confirm the fact; but at the ſame time ſhew that his hypotheſes were ill founded. He promiſes, however, to continue his attention to the ſubject, which is certainly a very intereſting one. In the courſe of his experinments Mr. Wilſon has diſcovered, that by mixing ſnow with alcohol, a great degree of cold is produced, though not equal to that which is produced by mixing it with ſpirit of nitre.

---

Mr. Thomas Hayes, ſurgeon at Hampſtead in Middleſex, having ſeen the article relative to Buxton, in our Journal for April, has favoured us with the following additional information concerning the waters of that place—" Formerly there was but one bath at Buxton, which ladies and gentlemen uſed at certain hours alternately, but at preſent there are two baths, one for the ladies ladies and the other for gentlemen, and both are large enough to hold, not four only, as the society have been informed, but ten or fourteen persons at the same time, very commodiously. Both baths are supplied by the same spring, and are continually filling and emptying, so that the waters are always fresh, and of the same temperature. The ladies have a room adjoining to their bath, but the gentlemen are obliged to dress and undress in the bathing room, which is very damp, from the vapour that is continually rising. This evil is justly complained of, and a room ought certainly to be erected for the gentlemen, as well as the ladies, as the cloaths get wet, and many inconveniences arise from the want of one. The price of bathing is a shilling for every person that does not lodge at the Hall where the baths are situated, but only six pence to those who do; but that every inn may be on a footing in this respect, Lord Scarsdale, who owns most of the other houses, returns, by the landlords of them, a sixpence to each person; and to make the accommodation compleat, a chair is provided by each landlord to carry his lodgers to the bath when it is wet, or they are unable to walk, and this without expence: but there is no priority or preference whatever in bathing, whether you lodge at the Hall or at the other inns, of which there are feveral very good ones, particularly the White Hart.

The heating the waters artificially, as mentioned in the Journal, would doubtlefs add greatly to their efficacy in many difeafes. I remember to have converfed with the late Dr. Fothergill on this fubject, who expreffed his hopes that the Duke of Devonfhire would add this to the many other improvements he is making at Buxton. That learned and ingenious phyfician, Dr. Percival, in his effay on thefe waters has very much recommended it; and, I may add, that the expence attending it would not be confifiderable, as coals are not dear there, and the artificial heat would be wanted only now and then.

The natural heat of the bath by Fahrenheit's thermometer is 82°. St. Ann's well, which is covered with a portico of ftone, but otherwife open to the common air, is 80°.

The waters by chymical analyfis feem to contain a fmall portion of foffil alkali, fea-falt, and calcareous earth, with a large quantity of fixable air; but there is a curative principle in them that no analogy can demonſtrate, or chymiſt properly define.

They prove ſingularly efficacious in chronic rheumatiſm, as I experienced lately in my own caſe. They are not recommended in the acute rheumatiſm, but if they were made hotter they would be very uſeful in that as well as in a variety of other diſeaſes in which they are not had recourſe to at preſent. I have ſeen the moſt obſtinate ſciaticas and rigidities of the ſpine cured by them, after every means under the direction of the moſt eminent phyſicians had failed. That worthy and judicious phyſician Dr. Bullock, who has attended the bath for many years, has accounts of their good effects in a great number of very intereſting caſes that have occurred in the courſe of his practice, which I hope he will publiſh, for it is a pity the public in general ſhould be deprived of ſuch uſeful information."

